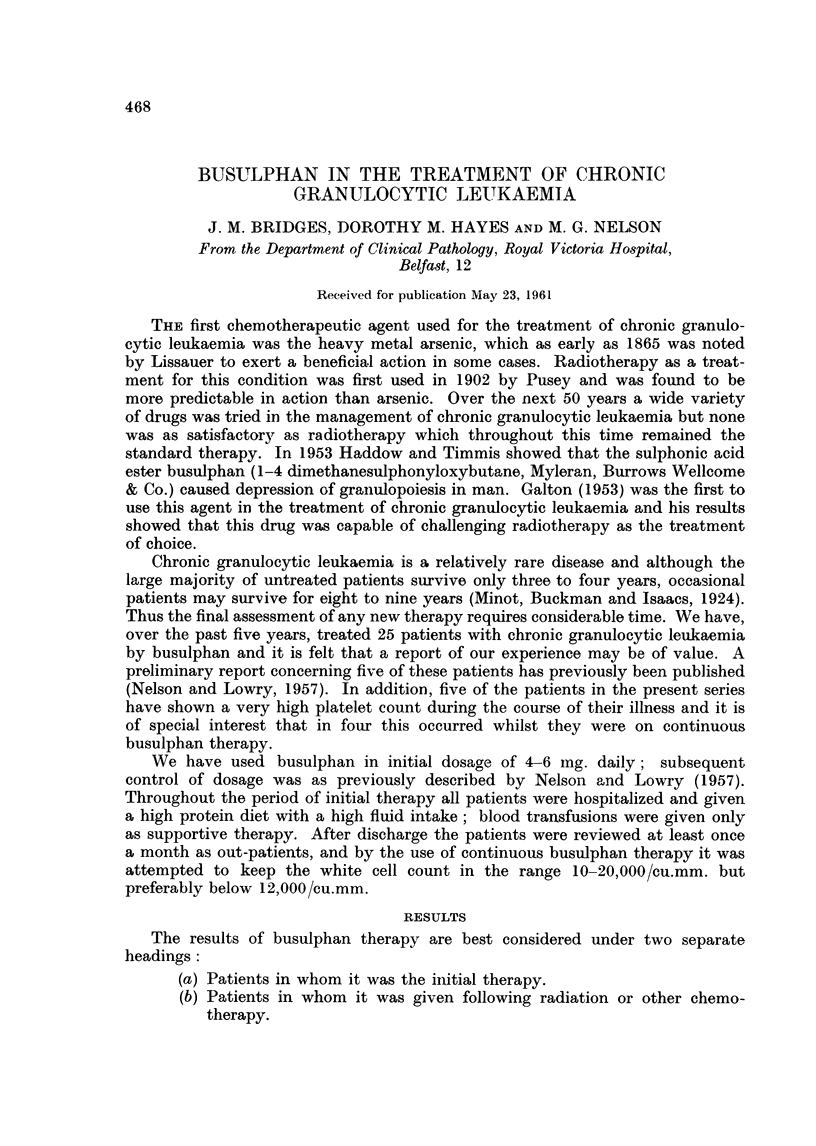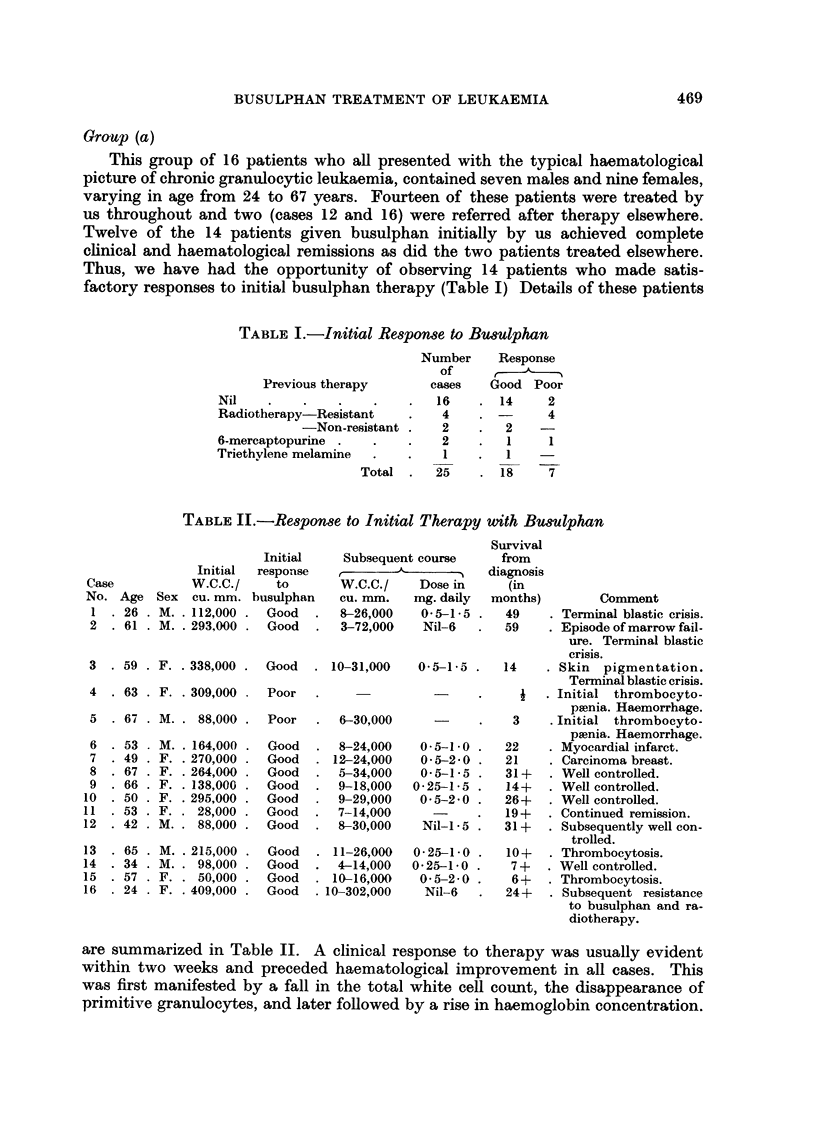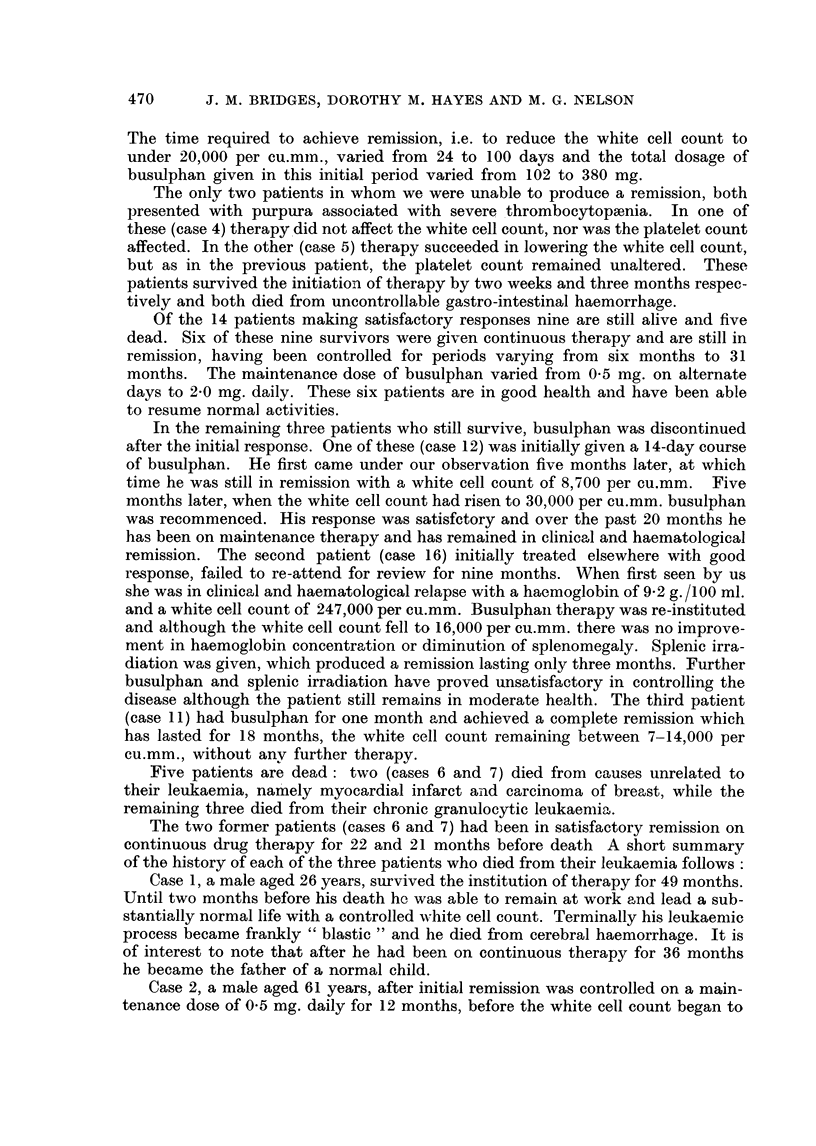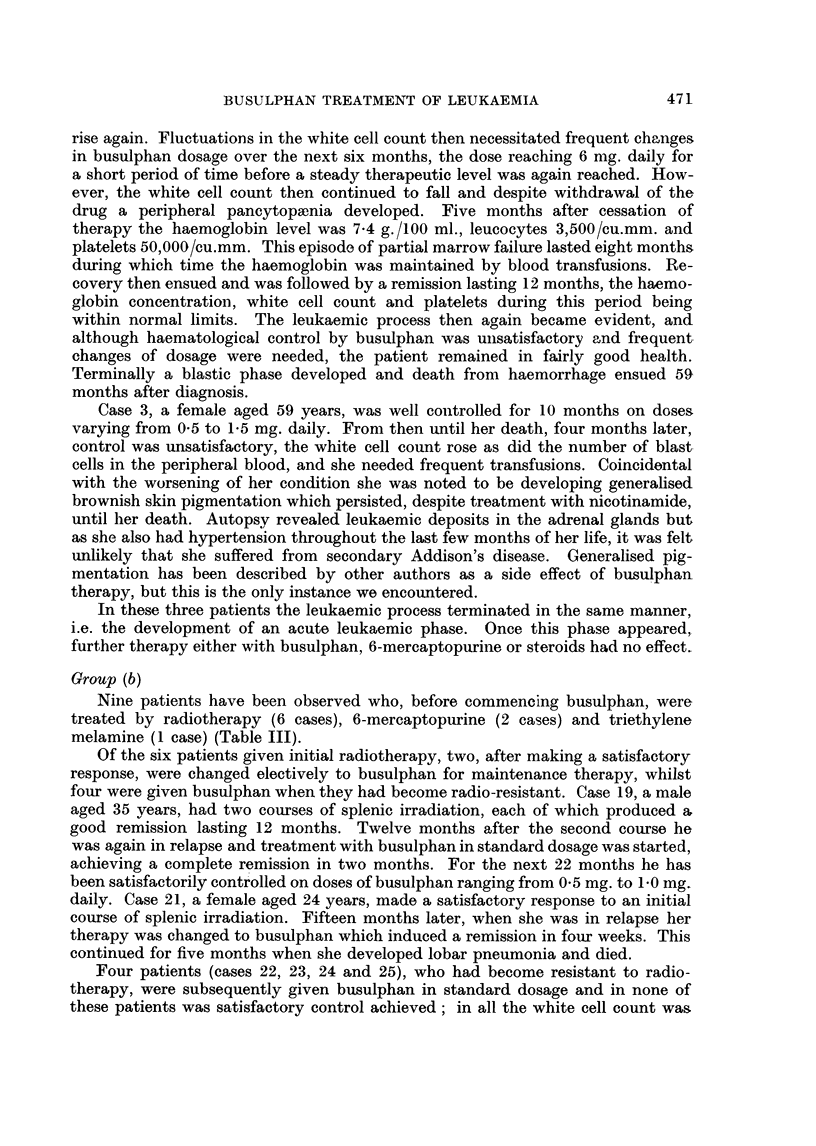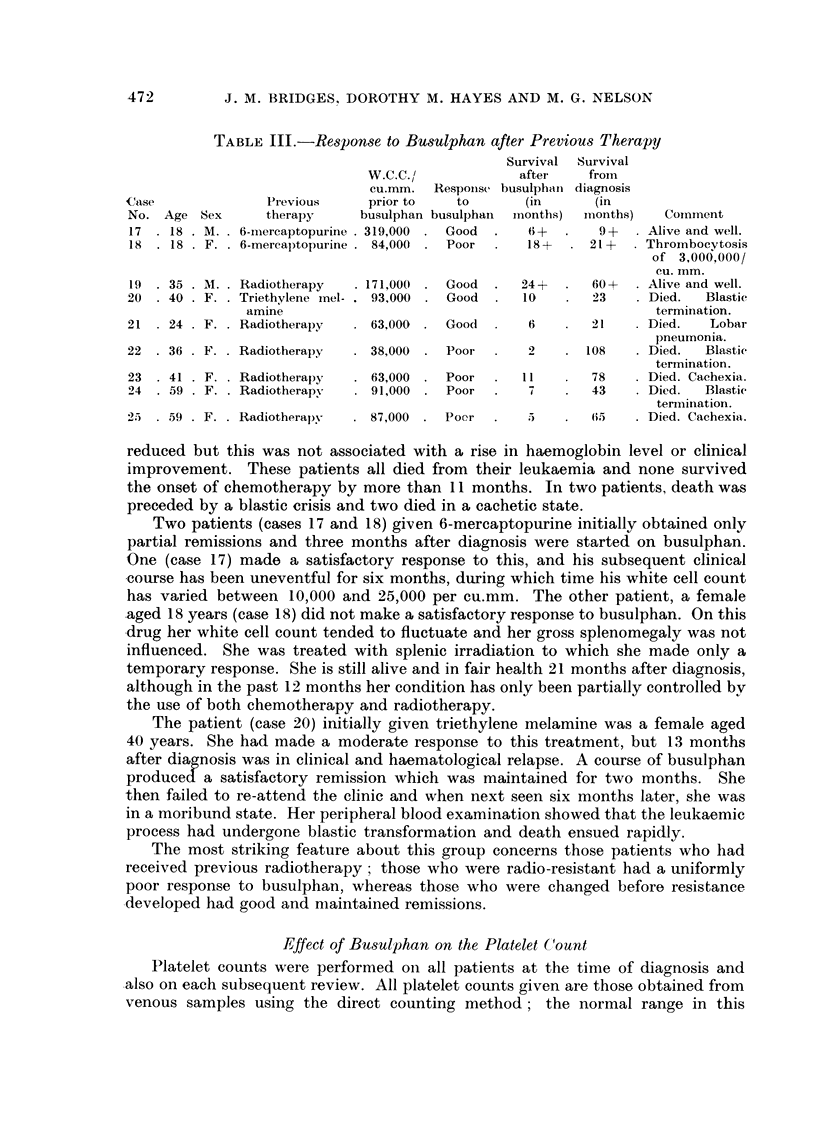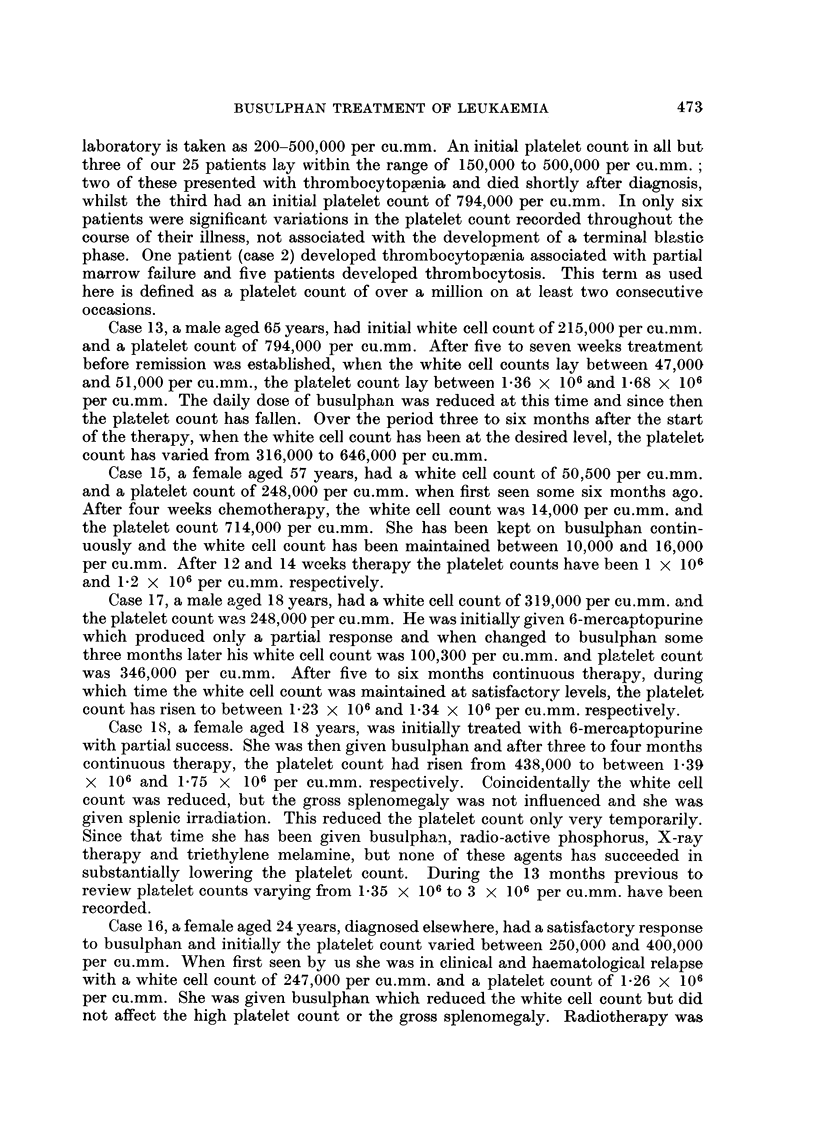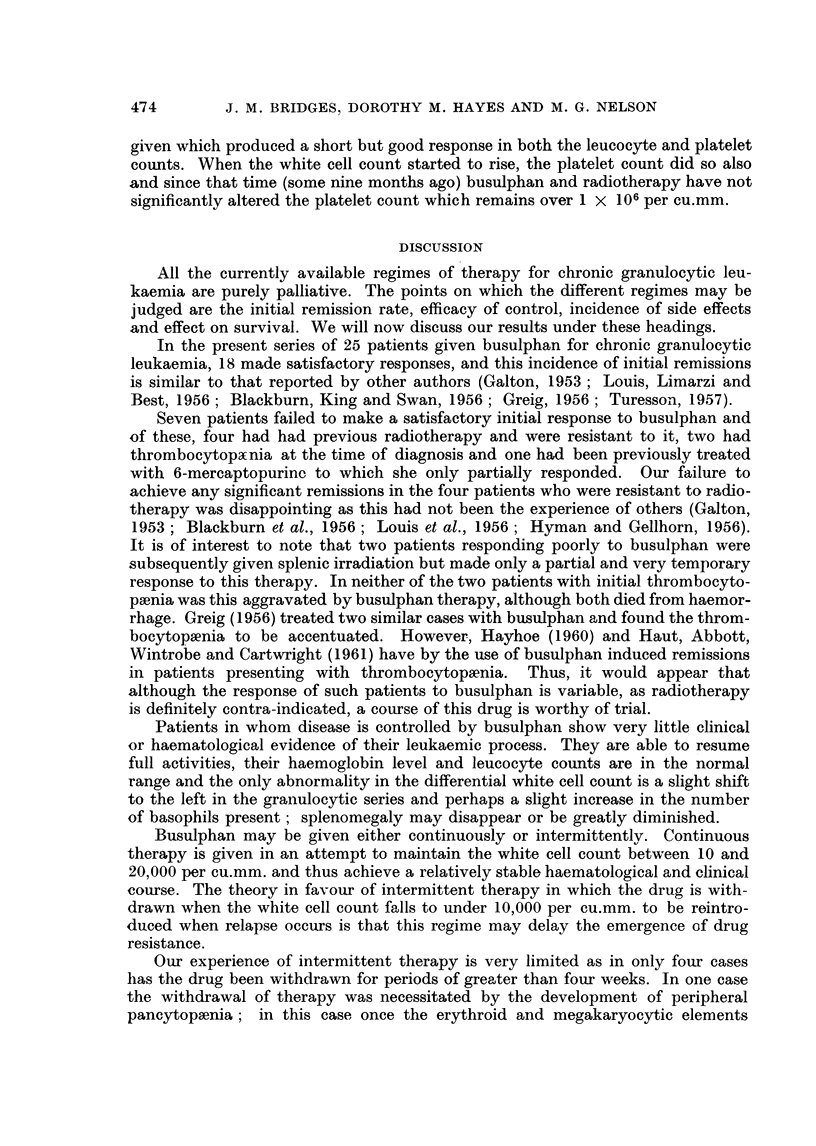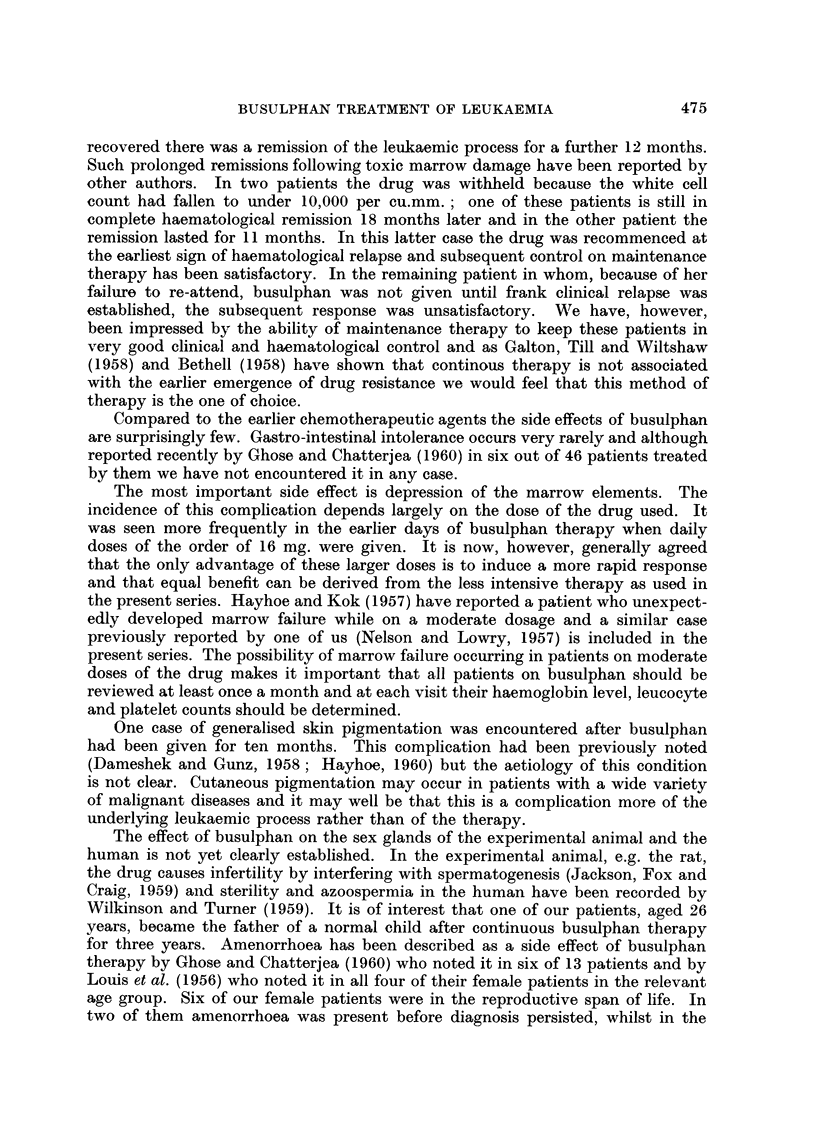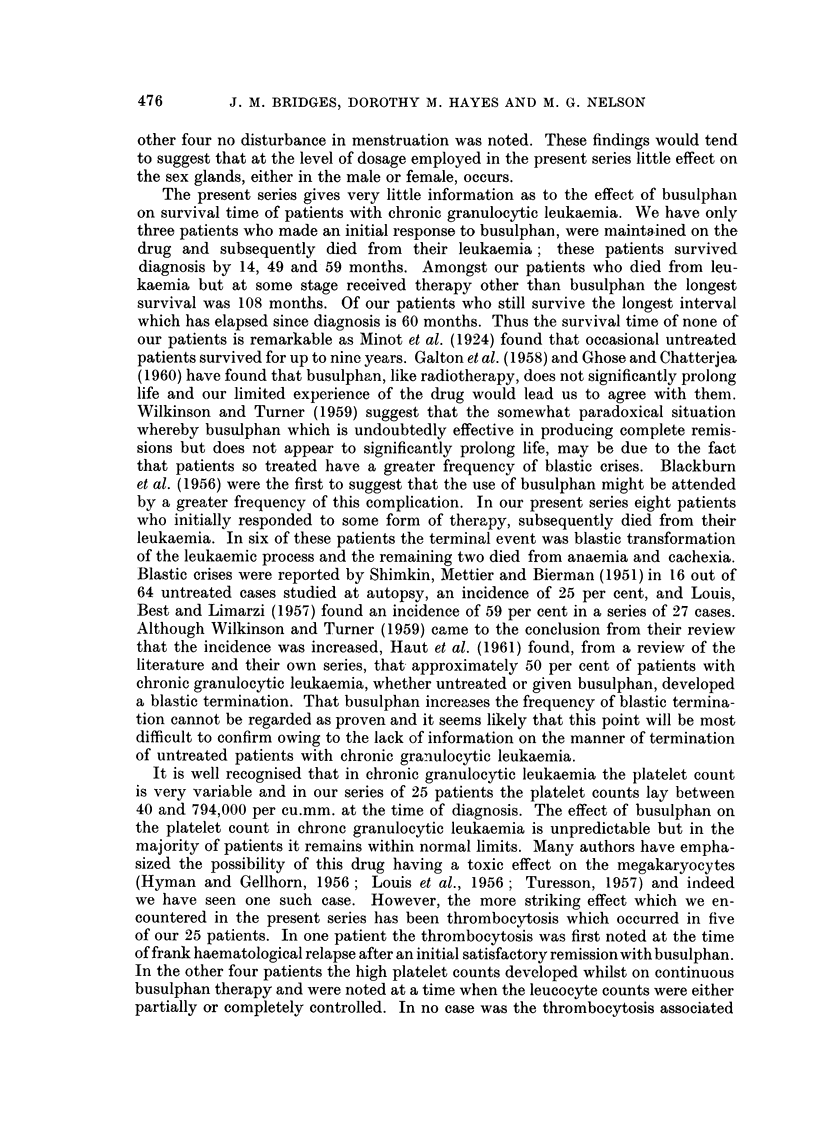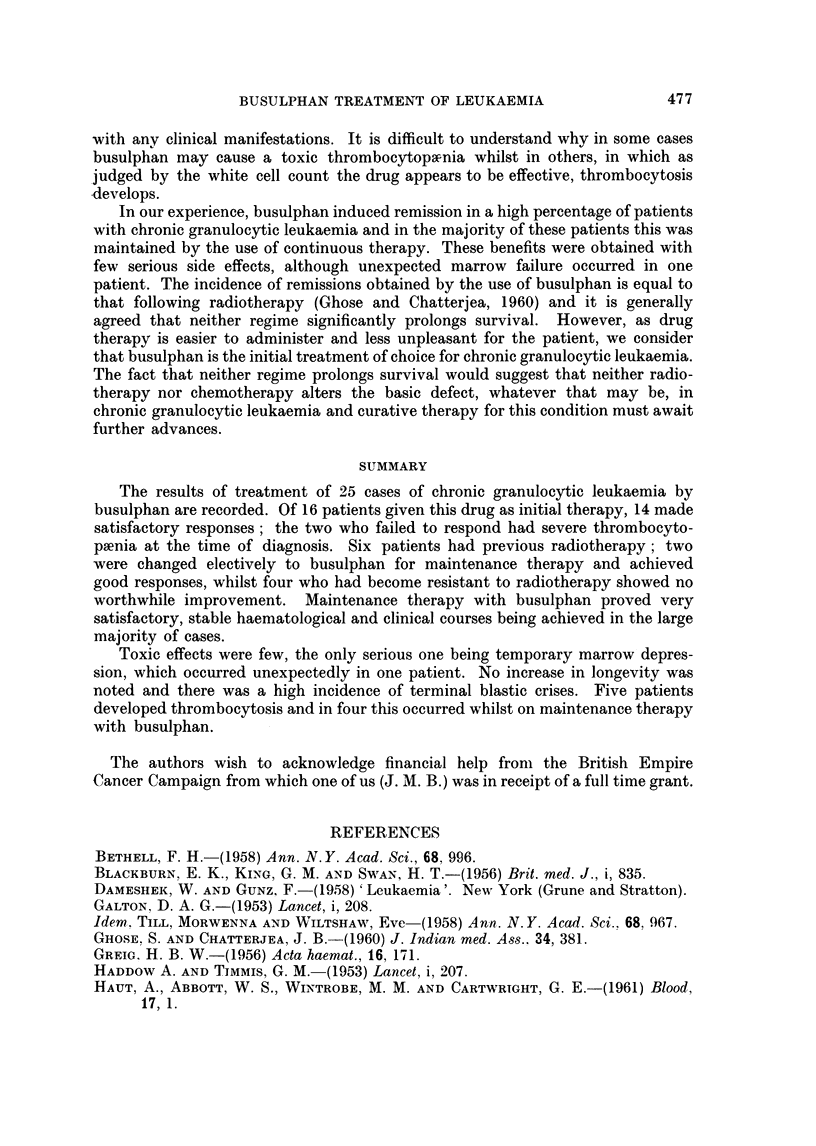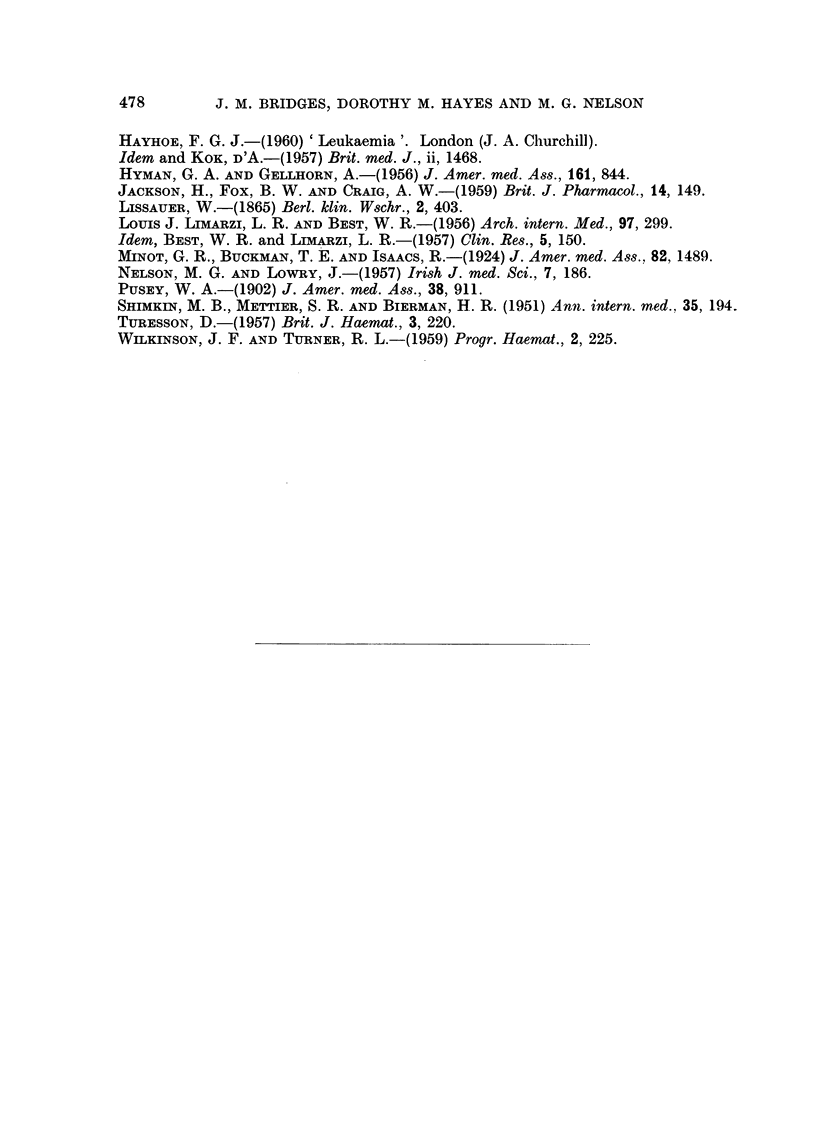# Busulphan in the Treatment of Chronic Granulocytic Leukaemia

**DOI:** 10.1038/bjc.1961.56

**Published:** 1961-09

**Authors:** J. M. Bridges, Dorothy M. Hayes, M. G. Nelson


### BUSULPHAN IN THE TREATMENT OF CHRONIC GRANULOCYTIC LEUKAEMIA

J. M. BRIDGES, DOROTHY M. HAYES AND M. G. NELSON

*From the Department of Clinical Pathology, Royal Victoria Hospital, Belfast, 12*

Received for publication May 23, 1961

THE first chemotherapeutic agent used for the treatment of chronic granulocytic leukaemia was the heavy metal arsenic, which as early as 1865 was noted by Lissauer to exert a beneficial action in some cases. Radiotherapy as a treatment for this condition was first used in 1902 by Pusey and was found to be more predictable in action than arsenic. Over the next 50 years a wide variety of drugs was tried in the management of chronic granulocytic leukaemia but none was as satisfactory as radiotherapy which throughout this time remained the standard therapy. In 1953 Haddow and Timmis showed that the sulphonic acid ester busulphan (1–4 dimethanesulphonyloxybutane, Myleran, Burrows Wellcome & Co.) caused depression of granulopoiesis in man. Galton (1953) was the first to use this agent in the treatment of chronic granulocytic leukaemia and his results showed that this drug was capable of challenging radiotherapy as the treatment of choice.

Chronic granulocytic leukaemia is a relatively rare disease and although the large majority of untreated patients survive only three to four years, occasional patients may survive for eight to nine years (Minot, Buckman and Isaacs, 1924). Thus the final assessment of any new therapy requires considerable time. We have, over the past five years, treated 25 patients with chronic granulocytic leukaemia by busulphan and it is felt that a report of our experience may be of value. A preliminary report concerning five of these patients has previously been published (Nelson and Lowry, 1957). In addition, five of the patients in the present series have shown a very high platelet count during the course of their illness and it is of special interest that in four this occurred whilst they were on continuous busulphan therapy.

We have used busulphan in initial dosage of 4–6 mg. daily ; subsequent control of dosage was as previously described by Nelson and Lowry (1957). Throughout the period of initial therapy all patients were hospitalized and given a high protein diet with a high fluid intake ; blood transfusions were given only as supportive therapy. After discharge the patients were reviewed at least once a month as out-patients, and by the use of continuous busulphan therapy it was attempted to keep the white cell count in the range 10–20,000/cu.mm. but preferably below 12,000/cu.mm.

### RESULTS

The results of busulphan therapy are best considered under two separate headings :

(*a*) Patients in whom it was the initial therapy.
(*b*) Patients in whom it was given following radiation or other chemotherapy.

*Group (a)*

This group of 16 patients who all presented with the typical haematological picture of chronic granulocytic leukaemia, contained seven males and nine females, varying in age from 24 to 67 years. Fourteen of these patients were treated by us throughout and two (cases 12 and 16) were referred after therapy elsewhere. Twelve of the 14 patients given busulphan initially by us achieved complete clinical and haematological remissions as did the two patients treated elsewhere. Thus, we have had the opportunity of observing 14 patients who made satisfactory responses to initial busulphan therapy (Table I) Details of these patients

TABLE I.—*Initial Response to Busulphan*

| Previous therapy | Number of cases | Response: Good | Response: Poor |
|---|---|---|---|
| Nil | 16 | 14 | 2 |
| Radiotherapy—Resistant | 4 | — | 4 |
| —Non-resistant | 2 | 2 | — |
| 6-mercaptopurine | 2 | 1 | 1 |
| Triethylene melamine | 1 | 1 | — |
| Total | 25 | 18 | 7 |

TABLE II.—*Response to Initial Therapy with Busulphan*

| Case No. | Age | Sex | Initial W.C.C./ cu. mm. | Initial response to busulphan | Subsequent course: W.C.C./ cu. mm. | Subsequent course: Dose in mg. daily | Survival from diagnosis (in months) | Comment |
|---|---|---|---|---|---|---|---|---|
| 1 | 26 | M. | 112,000 | Good | 8–26,000 | 0·5–1·5 | 49 | Terminal blastic crisis. |
| 2 | 61 | M. | 293,000 | Good | 3–72,000 | Nil–6 | 59 | Episode of marrow failure. Terminal blastic crisis. |
| 3 | 59 | F. | 338,000 | Good | 10–31,000 | 0·5–1·5 | 14 | Skin pigmentation. Terminal blastic crisis. |
| 4 | 63 | F. | 309,000 | Poor | — | — | ½ | Initial thrombocytopænia. Haemorrhage. |
| 5 | 67 | M. | 88,000 | Poor | 6–30,000 | — | 3 | Initial thrombocytopænia. Haemorrhage. |
| 6 | 53 | M. | 164,000 | Good | 8–24,000 | 0·5–1·0 | 22 | Myocardial infarct. |
| 7 | 49 | F. | 270,000 | Good | 12–24,000 | 0·5–2·0 | 21 | Carcinoma breast. |
| 8 | 67 | F. | 264,000 | Good | 5–34,000 | 0·5–1·5 | 31+ | Well controlled. |
| 9 | 66 | F. | 138,000 | Good | 9–18,000 | 0·25–1·5 | 14+ | Well controlled. |
| 10 | 50 | F. | 295,000 | Good | 9–29,000 | 0·5–2·0 | 26+ | Well controlled. |
| 11 | 53 | F. | 28,000 | Good | 7–14,000 | — | 19+ | Continued remission. |
| 12 | 42 | M. | 88,000 | Good | 8–30,000 | Nil–1·5 | 31+ | Subsequently well controlled. |
| 13 | 65 | M. | 215,000 | Good | 11–26,000 | 0·25–1·0 | 10+ | Thrombocytosis. |
| 14 | 34 | M. | 98,000 | Good | 4–14,000 | 0·25–1·0 | 7+ | Well controlled. |
| 15 | 57 | F. | 50,000 | Good | 10–16,000 | 0·5–2·0 | 6+ | Thrombocytosis. |
| 16 | 24 | F. | 409,000 | Good | 10–302,000 | Nil–6 | 24+ | Subsequent resistance to busulphan and radiotherapy. |

are summarized in Table II. A clinical response to therapy was usually evident within two weeks and preceded haematological improvement in all cases. This was first manifested by a fall in the total white cell count, the disappearance of primitive granulocytes, and later followed by a rise in haemoglobin concentration.

The time required to achieve remission, i.e. to reduce the white cell count to under 20,000 per cu.mm., varied from 24 to 100 days and the total dosage of busulphan given in this initial period varied from 102 to 380 mg.

The only two patients in whom we were unable to produce a remission, both presented with purpura associated with severe thrombocytopænia. In one of these (case 4) therapy did not affect the white cell count, nor was the platelet count affected. In the other (case 5) therapy succeeded in lowering the white cell count, but as in the previous patient, the platelet count remained unaltered. These patients survived the initiation of therapy by two weeks and three months respectively and both died from uncontrollable gastro-intestinal haemorrhage.

Of the 14 patients making satisfactory responses nine are still alive and five dead. Six of these nine survivors were given continuous therapy and are still in remission, having been controlled for periods varying from six months to 31 months. The maintenance dose of busulphan varied from 0·5 mg. on alternate days to 2·0 mg. daily. These six patients are in good health and have been able to resume normal activities.

In the remaining three patients who still survive, busulphan was discontinued after the initial response. One of these (case 12) was initially given a 14-day course of busulphan. He first came under our observation five months later, at which time he was still in remission with a white cell count of 8,700 per cu.mm. Five months later, when the white cell count had risen to 30,000 per cu.mm. busulphan was recommenced. His response was satisfctory and over the past 20 months he has been on maintenance therapy and has remained in clinical and haematological remission. The second patient (case 16) initially treated elsewhere with good response, failed to re-attend for review for nine months. When first seen by us she was in clinical and haematological relapse with a haemoglobin of 9·2 g./100 ml. and a white cell count of 247,000 per cu.mm. Busulphan therapy was re-instituted and although the white cell count fell to 16,000 per cu.mm. there was no improvement in haemoglobin concentration or diminution of splenomegaly. Splenic irradiation was given, which produced a remission lasting only three months. Further busulphan and splenic irradiation have proved unsatisfactory in controlling the disease although the patient still remains in moderate health. The third patient (case 11) had busulphan for one month and achieved a complete remission which has lasted for 18 months, the white cell count remaining between 7–14,000 per cu.mm., without any further therapy.

Five patients are dead : two (cases 6 and 7) died from causes unrelated to their leukaemia, namely myocardial infarct and carcinoma of breast, while the remaining three died from their chronic granulocytic leukaemia.

The two former patients (cases 6 and 7) had been in satisfactory remission on continuous drug therapy for 22 and 21 months before death A short summary of the history of each of the three patients who died from their leukaemia follows :

Case 1, a male aged 26 years, survived the institution of therapy for 49 months. Until two months before his death he was able to remain at work and lead a substantially normal life with a controlled white cell count. Terminally his leukaemic process became frankly " blastic " and he died from cerebral haemorrhage. It is of interest to note that after he had been on continuous therapy for 36 months he became the father of a normal child.

Case 2, a male aged 61 years, after initial remission was controlled on a maintenance dose of 0·5 mg. daily for 12 months, before the white cell count began to rise again. Fluctuations in the white cell count then necessitated frequent changes in busulphan dosage over the next six months, the dose reaching 6 mg. daily for a short period of time before a steady therapeutic level was again reached. However, the white cell count then continued to fall and despite withdrawal of the drug a peripheral pancytopænia developed. Five months after cessation of therapy the haemoglobin level was 7·4 g./100 ml., leucocytes 3,500/cu.mm. and platelets 50,000/cu.mm. This episode of partial marrow failure lasted eight months during which time the haemoglobin was maintained by blood transfusions. Recovery then ensued and was followed by a remission lasting 12 months, the haemoglobin concentration, white cell count and platelets during this period being within normal limits. The leukaemic process then again became evident, and although haematological control by busulphan was unsatisfactory and frequent changes of dosage were needed, the patient remained in fairly good health. Terminally a blastic phase developed and death from haemorrhage ensued 59 months after diagnosis.

Case 3, a female aged 59 years, was well controlled for 10 months on doses varying from 0·5 to 1·5 mg. daily. From then until her death, four months later, control was unsatisfactory, the white cell count rose as did the number of blast cells in the peripheral blood, and she needed frequent transfusions. Coincidental with the worsening of her condition she was noted to be developing generalised brownish skin pigmentation which persisted, despite treatment with nicotinamide, until her death. Autopsy revealed leukaemic deposits in the adrenal glands but as she also had hypertension throughout the last few months of her life, it was felt unlikely that she suffered from secondary Addison's disease. Generalised pigmentation has been described by other authors as a side effect of busulphan therapy, but this is the only instance we encountered.

In these three patients the leukaemic process terminated in the same manner, i.e. the development of an acute leukaemic phase. Once this phase appeared, further therapy either with busulphan, 6-mercaptopurine or steroids had no effect.

*Group* (*b*)

Nine patients have been observed who, before commencing busulphan, were treated by radiotherapy (6 cases), 6-mercaptopurine (2 cases) and triethylene melamine (1 case) (Table III).

Of the six patients given initial radiotherapy, two, after making a satisfactory response, were changed electively to busulphan for maintenance therapy, whilst four were given busulphan when they had become radio-resistant. Case 19, a male aged 35 years, had two courses of splenic irradiation, each of which produced a good remission lasting 12 months. Twelve months after the second course he was again in relapse and treatment with busulphan in standard dosage was started, achieving a complete remission in two months. For the next 22 months he has been satisfactorily controlled on doses of busulphan ranging from 0·5 mg. to 1·0 mg. daily. Case 21, a female aged 24 years, made a satisfactory response to an initial course of splenic irradiation. Fifteen months later, when she was in relapse her therapy was changed to busulphan which induced a remission in four weeks. This continued for five months when she developed lobar pneumonia and died.

Four patients (cases 22, 23, 24 and 25), who had become resistant to radiotherapy, were subsequently given busulphan in standard dosage and in none of these patients was satisfactory control achieved ; in all the white cell count was

TABLE III.—*Response to Busulphan after Previous Therapy*

| Case No. | Age | Sex | Previous therapy | W.C.C./ cu.mm. prior to busulphan | Response to busulphan | Survival after busulphan (in months) | Survival from diagnosis (in months) | Comment |
|---|---|---|---|---|---|---|---|---|
| 17 | 18 | M. | 6-mercaptopurine | 319,000 | Good | 6+ | 9+ | Alive and well. |
| 18 | 18 | F. | 6-mercaptopurine | 84,000 | Poor | 18+ | 21+ | Thrombocytosis of 3,000,000/ cu. mm. |
| 19 | 35 | M. | Radiotherapy | 171,000 | Good | 24+ | 60+ | Alive and well. |
| 20 | 40 | F. | Triethylene melamine | 93,000 | Good | 10 | 23 | Died. Blastic termination. |
| 21 | 24 | F. | Radiotherapy | 63,000 | Good | 6 | 21 | Died. Lobar pneumonia. |
| 22 | 36 | F. | Radiotherapy | 38,000 | Poor | 2 | 108 | Died. Blastic termination. |
| 23 | 41 | F. | Radiotherapy | 63,000 | Poor | 11 | 78 | Died. Cachexia. |
| 24 | 59 | F. | Radiotherapy | 91,000 | Poor | 7 | 43 | Died. Blastic termination. |
| 25 | 59 | F. | Radiotherapy | 87,000 | Poor | 5 | 65 | Died. Cachexia. |

reduced but this was not associated with a rise in haemoglobin level or clinical improvement. These patients all died from their leukaemia and none survived the onset of chemotherapy by more than 11 months. In two patients, death was preceded by a blastic crisis and two died in a cachetic state.

Two patients (cases 17 and 18) given 6-mercaptopurine initially obtained only partial remissions and three months after diagnosis were started on busulphan. One (case 17) made a satisfactory response to this, and his subsequent clinical course has been uneventful for six months, during which time his white cell count has varied between 10,000 and 25,000 per cu.mm. The other patient, a female aged 18 years (case 18) did not make a satisfactory response to busulphan. On this drug her white cell count tended to fluctuate and her gross splenomegaly was not influenced. She was treated with splenic irradiation to which she made only a temporary response. She is still alive and in fair health 21 months after diagnosis, although in the past 12 months her condition has only been partially controlled by the use of both chemotherapy and radiotherapy.

The patient (case 20) initially given triethylene melamine was a female aged 40 years. She had made a moderate response to this treatment, but 13 months after diagnosis was in clinical and haematological relapse. A course of busulphan produced a satisfactory remission which was maintained for two months. She then failed to re-attend the clinic and when next seen six months later, she was in a moribund state. Her peripheral blood examination showed that the leukaemic process had undergone blastic transformation and death ensued rapidly.

The most striking feature about this group concerns those patients who had received previous radiotherapy ; those who were radio-resistant had a uniformly poor response to busulphan, whereas those who were changed before resistance developed had good and maintained remissions.

### *Effect of Busulphan on the Platelet Count*

Platelet counts were performed on all patients at the time of diagnosis and also on each subsequent review. All platelet counts given are those obtained from venous samples using the direct counting method ; the normal range in this laboratory is taken as 200–500,000 per cu.mm. An initial platelet count in all but three of our 25 patients lay within the range of 150,000 to 500,000 per cu.mm.; two of these presented with thrombocytopænia and died shortly after diagnosis, whilst the third had an initial platelet count of 794,000 per cu.mm. In only six patients were significant variations in the platelet count recorded throughout the course of their illness, not associated with the development of a terminal blastic phase. One patient (case 2) developed thrombocytopænia associated with partial marrow failure and five patients developed thrombocytosis. This term as used here is defined as a platelet count of over a million on at least two consecutive occasions.

Case 13, a male aged 65 years, had initial white cell count of 215,000 per cu.mm. and a platelet count of 794,000 per cu.mm. After five to seven weeks treatment before remission was established, when the white cell counts lay between 47,000 and 51,000 per cu.mm., the platelet count lay between $1{\cdot}36 \times 10^6$ and $1{\cdot}68 \times 10^6$ per cu.mm. The daily dose of busulphan was reduced at this time and since then the platelet count has fallen. Over the period three to six months after the start of the therapy, when the white cell count has been at the desired level, the platelet count has varied from 316,000 to 646,000 per cu.mm.

Case 15, a female aged 57 years, had a white cell count of 50,500 per cu.mm. and a platelet count of 248,000 per cu.mm. when first seen some six months ago. After four weeks chemotherapy, the white cell count was 14,000 per cu.mm. and the platelet count 714,000 per cu.mm. She has been kept on busulphan continuously and the white cell count has been maintained between 10,000 and 16,000 per cu.mm. After 12 and 14 weeks therapy the platelet counts have been $1 \times 10^6$ and $1{\cdot}2 \times 10^6$ per cu.mm. respectively.

Case 17, a male aged 18 years, had a white cell count of 319,000 per cu.mm. and the platelet count was 248,000 per cu.mm. He was initially given 6-mercaptopurine which produced only a partial response and when changed to busulphan some three months later his white cell count was 100,300 per cu.mm. and platelet count was 346,000 per cu.mm. After five to six months continuous therapy, during which time the white cell count was maintained at satisfactory levels, the platelet count has risen to between $1{\cdot}23 \times 10^6$ and $1{\cdot}34 \times 10^6$ per cu.mm. respectively.

Case 18, a female aged 18 years, was initially treated with 6-mercaptopurine with partial success. She was then given busulphan and after three to four months continuous therapy, the platelet count had risen from 438,000 to between $1{\cdot}39 \times 10^6$ and $1{\cdot}75 \times 10^6$ per cu.mm. respectively. Coincidentally the white cell count was reduced, but the gross splenomegaly was not influenced and she was given splenic irradiation. This reduced the platelet count only very temporarily. Since that time she has been given busulphan, radio-active phosphorus, X-ray therapy and triethylene melamine, but none of these agents has succeeded in substantially lowering the platelet count. During the 13 months previous to review platelet counts varying from $1{\cdot}35 \times 10^6$ to $3 \times 10^6$ per cu.mm. have been recorded.

Case 16, a female aged 24 years, diagnosed elsewhere, had a satisfactory response to busulphan and initially the platelet count varied between 250,000 and 400,000 per cu.mm. When first seen by us she was in clinical and haematological relapse with a white cell count of 247,000 per cu.mm. and a platelet count of $1{\cdot}26 \times 10^6$ per cu.mm. She was given busulphan which reduced the white cell count but did not affect the high platelet count or the gross splenomegaly. Radiotherapy was given which produced a short but good response in both the leucocyte and platelet counts. When the white cell count started to rise, the platelet count did so also and since that time (some nine months ago) busulphan and radiotherapy have not significantly altered the platelet count which remains over 1 $\times$ $10^6$ per cu.mm.

### DISCUSSION

All the currently available regimes of therapy for chronic granulocytic leukaemia are purely palliative. The points on which the different regimes may be judged are the initial remission rate, efficacy of control, incidence of side effects and effect on survival. We will now discuss our results under these headings.

In the present series of 25 patients given busulphan for chronic granulocytic leukaemia, 18 made satisfactory responses, and this incidence of initial remissions is similar to that reported by other authors (Galton, 1953 ; Louis, Limarzi and Best, 1956 ; Blackburn, King and Swan, 1956 ; Greig, 1956 ; Turesson, 1957).

Seven patients failed to make a satisfactory initial response to busulphan and of these, four had had previous radiotherapy and were resistant to it, two had thrombocytopænia at the time of diagnosis and one had been previously treated with 6-mercaptopurinc to which she only partially responded. Our failure to achieve any significant remissions in the four patients who were resistant to radiotherapy was disappointing as this had not been the experience of others (Galton, 1953 ; Blackburn *et al.*, 1956 ; Louis *et al.*, 1956 ; Hyman and Gellhorn, 1956). It is of interest to note that two patients responding poorly to busulphan were subsequently given splenic irradiation but made only a partial and very temporary response to this therapy. In neither of the two patients with initial thrombocytopænia was this aggravated by busulphan therapy, although both died from haemorrhage. Greig (1956) treated two similar cases with busulphan and found the thrombocytopænia to be accentuated. However, Hayhoe (1960) and Haut, Abbott, Wintrobe and Cartwright (1961) have by the use of busulphan induced remissions in patients presenting with thrombocytopænia. Thus, it would appear that although the response of such patients to busulphan is variable, as radiotherapy is definitely contra-indicated, a course of this drug is worthy of trial.

Patients in whom disease is controlled by busulphan show very little clinical or haematological evidence of their leukaemic process. They are able to resume full activities, their haemoglobin level and leucocyte counts are in the normal range and the only abnormality in the differential white cell count is a slight shift to the left in the granulocytic series and perhaps a slight increase in the number of basophils present ; splenomegaly may disappear or be greatly diminished.

Busulphan may be given either continuously or intermittently. Continuous therapy is given in an attempt to maintain the white cell count between 10 and 20,000 per cu.mm. and thus achieve a relatively stable haematological and clinical course. The theory in favour of intermittent therapy in which the drug is withdrawn when the white cell count falls to under 10,000 per cu.mm. to be reintroduced when relapse occurs is that this regime may delay the emergence of drug resistance.

Our experience of intermittent therapy is very limited as in only four cases has the drug been withdrawn for periods of greater than four weeks. In one case the withdrawal of therapy was necessitated by the development of peripheral pancytopænia ; in this case once the erythroid and megakaryocytic elements recovered there was a remission of the leukaemic process for a further 12 months. Such prolonged remissions following toxic marrow damage have been reported by other authors. In two patients the drug was withheld because the white cell count had fallen to under 10,000 per cu.mm. ; one of these patients is still in complete haematological remission 18 months later and in the other patient the remission lasted for 11 months. In this latter case the drug was recommenced at the earliest sign of haematological relapse and subsequent control on maintenance therapy has been satisfactory. In the remaining patient in whom, because of her failure to re-attend, busulphan was not given until frank clinical relapse was established, the subsequent response was unsatisfactory. We have, however, been impressed by the ability of maintenance therapy to keep these patients in very good clinical and haematological control and as Galton, Till and Wiltshaw (1958) and Bethell (1958) have shown that continous therapy is not associated with the earlier emergence of drug resistance we would feel that this method of therapy is the one of choice.

Compared to the earlier chemotherapeutic agents the side effects of busulphan are surprisingly few. Gastro-intestinal intolerance occurs very rarely and although reported recently by Ghose and Chatterjea (1960) in six out of 46 patients treated by them we have not encountered it in any case.

The most important side effect is depression of the marrow elements. The incidence of this complication depends largely on the dose of the drug used. It was seen more frequently in the earlier days of busulphan therapy when daily doses of the order of 16 mg. were given. It is now, however, generally agreed that the only advantage of these larger doses is to induce a more rapid response and that equal benefit can be derived from the less intensive therapy as used in the present series. Hayhoe and Kok (1957) have reported a patient who unexpectedly developed marrow failure while on a moderate dosage and a similar case previously reported by one of us (Nelson and Lowry, 1957) is included in the present series. The possibility of marrow failure occurring in patients on moderate doses of the drug makes it important that all patients on busulphan should be reviewed at least once a month and at each visit their haemoglobin level, leucocyte and platelet counts should be determined.

One case of generalised skin pigmentation was encountered after busulphan had been given for ten months. This complication had been previously noted (Dameshek and Gunz, 1958 ; Hayhoe, 1960) but the aetiology of this condition is not clear. Cutaneous pigmentation may occur in patients with a wide variety of malignant diseases and it may well be that this is a complication more of the underlying leukaemic process rather than of the therapy.

The effect of busulphan on the sex glands of the experimental animal and the human is not yet clearly established. In the experimental animal, e.g. the rat, the drug causes infertility by interfering with spermatogenesis (Jackson, Fox and Craig, 1959) and sterility and azoospermia in the human have been recorded by Wilkinson and Turner (1959). It is of interest that one of our patients, aged 26 years, became the father of a normal child after continuous busulphan therapy for three years. Amenorrhoea has been described as a side effect of busulphan therapy by Ghose and Chatterjea (1960) who noted it in six of 13 patients and by Louis *et al.* (1956) who noted it in all four of their female patients in the relevant age group. Six of our female patients were in the reproductive span of life. In two of them amenorrhoea was present before diagnosis persisted, whilst in the other four no disturbance in menstruation was noted. These findings would tend to suggest that at the level of dosage employed in the present series little effect on the sex glands, either in the male or female, occurs.

The present series gives very little information as to the effect of busulphan on survival time of patients with chronic granulocytic leukaemia. We have only three patients who made an initial response to busulphan, were maintained on the drug and subsequently died from their leukaemia; these patients survived diagnosis by 14, 49 and 59 months. Amongst our patients who died from leukaemia but at some stage received therapy other than busulphan the longest survival was 108 months. Of our patients who still survive the longest interval which has elapsed since diagnosis is 60 months. Thus the survival time of none of our patients is remarkable as Minot *et al.* (1924) found that occasional untreated patients survived for up to nine years. Galton *et al.* (1958) and Ghose and Chatterjea (1960) have found that busulphan, like radiotherapy, does not significantly prolong life and our limited experience of the drug would lead us to agree with them. Wilkinson and Turner (1959) suggest that the somewhat paradoxical situation whereby busulphan which is undoubtedly effective in producing complete remissions but does not appear to significantly prolong life, may be due to the fact that patients so treated have a greater frequency of blastic crises. Blackburn *et al.* (1956) were the first to suggest that the use of busulphan might be attended by a greater frequency of this complication. In our present series eight patients who initially responded to some form of therapy, subsequently died from their leukaemia. In six of these patients the terminal event was blastic transformation of the leukaemic process and the remaining two died from anaemia and cachexia. Blastic crises were reported by Shimkin, Mettier and Bierman (1951) in 16 out of 64 untreated cases studied at autopsy, an incidence of 25 per cent, and Louis, Best and Limarzi (1957) found an incidence of 59 per cent in a series of 27 cases. Although Wilkinson and Turner (1959) came to the conclusion from their review that the incidence was increased, Haut *et al.* (1961) found, from a review of the literature and their own series, that approximately 50 per cent of patients with chronic granulocytic leukaemia, whether untreated or given busulphan, developed a blastic termination. That busulphan increases the frequency of blastic termination cannot be regarded as proven and it seems likely that this point will be most difficult to confirm owing to the lack of information on the manner of termination of untreated patients with chronic granulocytic leukaemia.

It is well recognised that in chronic granulocytic leukaemia the platelet count is very variable and in our series of 25 patients the platelet counts lay between 40 and 794,000 per cu.mm. at the time of diagnosis. The effect of busulphan on the platelet count in chronc granulocytic leukaemia is unpredictable but in the majority of patients it remains within normal limits. Many authors have emphasized the possibility of this drug having a toxic effect on the megakaryocytes (Hyman and Gellhorn, 1956; Louis *et al.*, 1956; Turesson, 1957) and indeed we have seen one such case. However, the more striking effect which we encountered in the present series has been thrombocytosis which occurred in five of our 25 patients. In one patient the thrombocytosis was first noted at the time of frank haematological relapse after an initial satisfactory remission with busulphan. In the other four patients the high platelet counts developed whilst on continuous busulphan therapy and were noted at a time when the leucocyte counts were either partially or completely controlled. In no case was the thrombocytosis associated with any clinical manifestations. It is difficult to understand why in some cases busulphan may cause a toxic thrombocytopænia whilst in others, in which as judged by the white cell count the drug appears to be effective, thrombocytosis develops.

In our experience, busulphan induced remission in a high percentage of patients with chronic granulocytic leukaemia and in the majority of these patients this was maintained by the use of continuous therapy. These benefits were obtained with few serious side effects, although unexpected marrow failure occurred in one patient. The incidence of remissions obtained by the use of busulphan is equal to that following radiotherapy (Ghose and Chatterjea, 1960) and it is generally agreed that neither regime significantly prolongs survival. However, as drug therapy is easier to administer and less unpleasant for the patient, we consider that busulphan is the initial treatment of choice for chronic granulocytic leukaemia. The fact that neither regime prolongs survival would suggest that neither radiotherapy nor chemotherapy alters the basic defect, whatever that may be, in chronic granulocytic leukaemia and curative therapy for this condition must await further advances.

### SUMMARY

The results of treatment of 25 cases of chronic granulocytic leukaemia by busulphan are recorded. Of 16 patients given this drug as initial therapy, 14 made satisfactory responses ; the two who failed to respond had severe thrombocytopænia at the time of diagnosis. Six patients had previous radiotherapy ; two were changed electively to busulphan for maintenance therapy and achieved good responses, whilst four who had become resistant to radiotherapy showed no worthwhile improvement. Maintenance therapy with busulphan proved very satisfactory, stable haematological and clinical courses being achieved in the large majority of cases.

Toxic effects were few, the only serious one being temporary marrow depression, which occurred unexpectedly in one patient. No increase in longevity was noted and there was a high incidence of terminal blastic crises. Five patients developed thrombocytosis and in four this occurred whilst on maintenance therapy with busulphan.

The authors wish to acknowledge financial help from the British Empire Cancer Campaign from which one of us (J. M. B.) was in receipt of a full time grant.

### REFERENCES

BETHELL, F. H.—(1958) *Ann. N.Y. Acad. Sci.*, **68**, 996.
BLACKBURN, E. K., KING, G. M. AND SWAN, H. T.—(1956) *Brit. med. J.*, i, 835.
DAMESHEK, W. AND GUNZ, F.—(1958) 'Leukaemia'. New York (Grune and Stratton).
GALTON, D. A. G.—(1953) *Lancet*, i, 208.
*Idem*, TILL, MORWENNA AND WILTSHAW, Eve—(1958) *Ann. N.Y. Acad. Sci.*, **68**, 967.
GHOSE, S. AND CHATTERJEA, J. B.—(1960) *J. Indian med. Ass.*, **34**, 381.
GREIG, H. B. W.—(1956) *Acta haemat.*, **16**, 171.
HADDOW A. AND TIMMIS, G. M.—(1953) *Lancet*, i, 207.
HAUT, A., ABBOTT, W. S., WINTROBE, M. M. AND CARTWRIGHT, G. E.—(1961) *Blood*, **17**, 1.

HAYHOE, F. G. J.—(1960) ' Leukaemia '. London (J. A. Churchill).
*Idem* and KOK, D'A.—(1957) *Brit. med. J.*, ii, 1468.
HYMAN, G. A. AND GELLHORN, A.—(1956) *J. Amer. med. Ass.*, **161**, 844.
JACKSON, H., FOX, B. W. AND CRAIG, A. W.—(1959) *Brit. J. Pharmacol.*, **14**, 149.
LISSAUER, W.—(1865) *Berl. klin. Wschr.*, **2**, 403.
LOUIS J. LIMARZI, L. R. AND BEST, W. R.—(1956) *Arch. intern. Med.*, **97**, 299.
*Idem*, BEST, W. R. and LIMARZI, L. R.—(1957) *Clin. Res.*, **5**, 150.
MINOT, G. R., BUCKMAN, T. E. AND ISAACS, R.—(1924) *J. Amer. med. Ass.*, **82**, 1489.
NELSON, M. G. AND LOWRY, J.—(1957) *Irish J. med. Sci.*, **7**, 186.
PUSEY, W. A.—(1902) *J. Amer. med. Ass.*, **38**, 911.
SHIMKIN, M. B., METTIER, S. R. AND BIERMAN, H. R. (1951) *Ann. intern. med.*, **35**, 194.
TURESSON, D.—(1957) *Brit. J. Haemat.*, **3**, 220.
WILKINSON, J. F. AND TURNER, R. L.—(1959) *Progr. Haemat.*, **2**, 225.